# Tracking a Fast-Moving Disease: Longitudinal Markers, Monitoring, and Clinical Trial Endpoints in ALS

**DOI:** 10.3389/fneur.2019.00229

**Published:** 2019-03-19

**Authors:** Rangariroyashe Hannah Chipika, Eoin Finegan, Stacey Li Hi Shing, Orla Hardiman, Peter Bede

**Affiliations:** Computational Neuroimaging Group, Biomedical Sciences Institute, Trinity College Dublin, Dublin, Ireland

**Keywords:** motor neuron disease, amyotrophic lateral sclerosis, biomarkers, magnetic resonance imaging, neuroimaging

## Abstract

Amyotrophic lateral sclerosis (ALS) encompasses a heterogeneous group of phenotypes with different progression rates, varying degree of extra-motor involvement and divergent progression patterns. The natural history of ALS is increasingly evaluated by large, multi-time point longitudinal studies, many of which now incorporate presymptomatic and post-mortem assessments. These studies not only have the potential to characterize patterns of anatomical propagation, molecular mechanisms of disease spread, but also to identify pragmatic monitoring markers. Sensitive markers of progressive neurodegenerative change are indispensable for clinical trials and individualized patient care. Biofluid markers, neuroimaging indices, electrophysiological markers, rating scales, questionnaires, and other disease-specific instruments have divergent sensitivity profiles. The discussion of candidate monitoring markers in ALS has a dual academic and clinical relevance, and is particularly timely given the increasing number of pharmacological trials. The objective of this paper is to provide a comprehensive and critical review of longitudinal studies in ALS, focusing on the sensitivity profile of established and emerging monitoring markers.

## Introduction

Amyotrophic lateral sclerosis (ALS) is a clinically, genetically, and pathologically heterogeneous neurodegenerative condition (1–3). Clinical heterogeneity in ALS is multidimensional owing to variations in upper motor neuron (UMN) and lower motor neuron (LMN) involvement, extra-motor symptoms, age of onset, survival, and progression-rates. Disease heterogeneity hinders biomarker development (3, 4) which in turn impedes the reliable assessment of candidate drugs in clinical trials (1). Current clinical trials recruit relatively heterogeneous cohorts of symptomatic patients, despite the notion that considerable pathological changes can already be detected at the time of diagnosis (5, 6). The considerable variability in progression rates in ALS is another confounding factor in clinical trial designs (1, 7–10). Imaging and electrophysiological markers have been repeatedly proposed as candidate monitoring markers (11, 12), but it is increasingly clear that a panel of several “wet” and “dry” biomarkers may be required to capture subtle changes over short periods of time (13, 14). The objective of this paper is the comprehensive and critical review of longitudinal studies in ALS, focusing on study designs, statistical power, clinical correlations, the sensitivity profile of proposed monitoring markers and their applicability to clinical trials.

## Methods

A formal literature search was performed on PubMed using the core search terms “amyotrophic lateral sclerosis” and “longitudinal” combined with each of the following keywords separately: “staging,” “monitoring,” “outcomes,” “clinical,” “clinical trials,” “electrophysiology,” “neurophysiology,” “electromyography,” “transcranial magnetic stimulation,” “motor unit number estimation,” “motor unit number index,” “positon emission tomography,” “single photon emission computed tomography,” “magnetic resonance imaging,” “neuroimaging,” “imaging,” “blood,” “urine,” “cerebrospinal fluid,” “saliva,” and “muscle.” A supplementary search combined the core search terms with the following keywords: “presymptomatic,” “asymptomatic,” and “post-mortem.” Inclusion criteria included longitudinal studies investigating imaging, neurophysiological, clinical, or biofluid biomarkers in ALS. Animal studies, review papers, opinion pieces, editorials, case reports, and case series were excluded. Only articles written in English and published between January 1980 and August 2018 were reviewed. Based on the above criteria a total of 118 original research papers were selected and reviewed in accordance with the Preferred Reporting Items for Systematic Reviews and Meta-Analyses (PRISMA) recommendations.

## Results

### Neuroimaging

The sample size characteristics, study design features, follow-up intervals of longitudinal neuroimaging, neurophysiology, and clinical studies are summarized in Table 1. Whilst most longitudinal imaging studies in ALS evaluate cerebral alterations (10), a number of promising spinal studies have now also been published. Spinal imaging has gradually overcome the technical challenges of physiological motion, small cross-sectional dimensions and susceptibility gradients (19, 110–118). The majority of longitudinal studies in ALS are single-center studies eliminating the need for cross-platform MR sequence harmonization and inter-rater reliability tests. Given the low incidence of certain phenotypes such as primary lateral sclerosis (PLS), progressive muscular atrophy (PMA), and spinal and bulbar muscular atrophy (SBMA) however, multisite collaboration is often necessary (119). The infrastructure, funding and governance of such multicenter collaborations are now established via international consortia like the Neuroimaging Society in Amyotrophic Lateral Sclerosis (NISALS) or the Northeast ALS Consortium (NEALS) (16, 23, 120, 121). The need to include disease-controls in addition to healthy controls to describe ALS-specific changes is increasingly recognized (30, 43, 44). With few exceptions (122–124), most ALS imaging studies use 3 Tesla platforms and 7 Tesla systems are more commonly used in post-mortem studies (125, 126). Disease progression has been detected across a range of MR imaging metrics including structural (22, 26), diffusion (16, 18), functional (28, 40), and spectroscopy (41, 42) measures. As the majority of studies have a two-timepoint design, it is often unclear if specific imaging metrics show linear or exponential changes. The few existing multi-timepoint studies suggest that pathological change is not linear (10). The revised ALS functional rating scale (ALSFRS-r) is the most commonly reported clinical measure (16, 18–20), with only few imaging studies reporting associations with staging (15) or neuropsychological performance (15, 24).

Table 1Longitudinal “dry biomarker” studies in ALS: Neuroimaging, Neurophysiology and Clinical Studies.

**Table:** 

**Author(s) and year of publication**	**Follow-up interval (months)**	**Number of patients/Number or controls**	**Clinical assessment batteries/Functional rating scales**	**Imaging data**	**Main study findings**
**IMAGING STUDIES**					
Floeter et al. (15)	6–18	28/28	ALSFRS-R, letter fluency, FBI, MMSE	DWI, structural (T2)	- progression and propagation detected (DTI measures) over 6 months - DTI measures correlated with ALSFRS-R, King's stage and cognitive measures
Kassubek et al. (16)	6	67/31	ALSFRS-R	DTI	- progression detected at group level and 27% of individual patients (DTI measures) - FA correlated with ALSFRS-R
Stampfli et al. (17)	3–6	21/13	ALSFRS-R	T1, DWI	-progression detected (FD values)
Baldaranov et al. (18)	26	6/6	ALSFRS-R	DTI	-progression detected (FA, AD/RD values) and correlated with progression on ALSFRS-R
Bede et al., 2017 (14)	4	32/69	ALSFRS-R	structural,DTI	-progression detected (GM)
de Albuquerque et al. (19)	8	27/27	ALSFRS-R, UMN scale	structural (T1, T2)	- progression detected (AD, MD) - correlation with ALSFRS-R change
Menke et al. (20)	24	16/0	ALSFRS-R, UMN score	T1, DTI, rs-Fmri	- progression detected - correlation with ALSFRS-R decline
Simon et al. (21)	3–6	21/13	ALSFRS-R, MRCSS-LL, MUNE	DTI, structural (T1)	- progression detected (FA values) - correlations with ALSFRS-R change, MUNE, functional disability and strength
Floeter et al. (22)	6	49/28	ALSFRS-R, FBI, MDRS-2, letter fluency, MMSE, D-KEFS	structural (T1)	-progression detected (ventricular volume)
Schulthess et al. (23)	6	135/56	ALSFRS-R	rs-Fmri, DTI	- progression detected (functional connectivity) - correlation with physical disability
McMillan et al. (24)	12	20/25	neuropsychology	structural (T1)	-hypermethylation protective against progression, correlation with protection of some components of neuropsychological assessment
Steinbach et al. (25)	3	16/16	ALSFRS-R, neuropsychology	DTI	-progression detected
Westeneng et al. (26)	5.5	112/60	ALSFRS-R	structural (T1)	- progression detected (volume measures) - correlation with ALSFRS-R
Menke et al. (4)	6	60/36	ALSFRS-R, ACE-R	structural (T1), DTI	-progression detected (GM)
Schuster et al. (27)	3–15	77/60	ALSFRS-R	structural (T1)	-progression detected (cortical thickness)
Stoppel et al. (28)	3	40/42	ALSFRS-R, MRC, neuropsychology	structural, Fmri	- progression detected - correlation with ALSFRS-R and MRC
Verstraete et al. (29)	5.5	24/19	ALSFRS-R	DTI, structural (T1)	- no progression detected - propagation detected
Ignjatovic et al. (30)	6	46/26	ALSFRS-R	structural (T1, T2, FLAIR)	-progression detected (hypointensities in PGGM)
Kwan et al. (31)	1.26–2.08 years	45/19	ALSFRS-R, finger tapping	T1, DTI	-progression detected (cortical thickness, GM volume)
Keil et al. (32)	6	24/24	ALSFRS-R, SF36, FAB, MMSE	DTI, structural (T1, T2)	- progression detected (FA values) - correlations with ALSFRS-R, physical and executive function
Menke et al. (33)	6	24/0	ALSFRS-R	DTI	-progression detected (AD)
Ichikawa et al. (34)	NA	6/NA	NA	NA	-progression detected, correlated to neuropsychology assessment
van der Graaff et al. (35)	NA	48/12	ALSFRS-R, finger tapping	DWI	-progression detected
Zhang et al. (36)	8	17/19	ALSFRS-R	structural (T1), DTI	- progression detected (FA)
Agosta et al. (37)	9	16/10	ALSFRS	structural (T1)	- progression detected (GM)
Agosta et al. (38)	9	17/20	ALSFRS	DWI, structural	- progression detected (cord area, cord average FA)
Avants et al. (39)	5.3	4/4	0	structural (T1)	- progression detected (cortical atrophy)
Lule et al. (40)	6	25/15	ALSFRS-R	Fmri, structural (T1)	- progression detected (activity)
Unrath et al. (41)	6	11/0	ALSFRS	MRS, T1	- progression detected (NAA, NAA/Cr+Cho)
Suhy et al. (42)	Every 3 months	28/12	0	MRS, T1, T2	- progression detected (NAA, Cr, Cho)
Block et al. (43)	24	33/20	0	MRS	- progression detected
Irwin et al. (44)		143/0	MMSE, LGVF	structural VBM	- no progression on MRI reported
Kolind et al. (45)	42	30/12	ALSFRS-R, ACE,	mcDESPOT	- progression detected in PLS only
Verstraete et al. (46)	6	45/25	ALSFRS-R	structural (T1)	- no progression reported
Blain et al. (47)	6–12	23/25	ALSFRS-R, ALSS	structural (T2), DWI	- no significant progression detected (DTI measures)
Rule et al. (48)	3–12	45/17	0	MRS, structural (T1, T2)	- no clear pattern of progressive change over time (NAA rations)
**Author(s) and year of publication**	**Follow-up interval (months)**	**Total number of patients/Total number of controls**	**Neurophysiology modality**	**Target muscle**	**Key study findings**
**NEUROPHYSIOLOGY STUDIES**					
Escorcio-Bezerra et al. (49)	4.3	21/21	MUNIX	tibialis anterior (TA), abductor pollicis brevis (APB) and abductor digiti minimi (ADM) muscles	- progression detected (mean MUNIX)
de Carvalho et al. (50)	3–6	73/37	FPs, MUPs, fibs-sw, jitter- MU physiology	tibialis anterior	- progression detected
Boekestein et al. (51)	8	18/24	MUNIX, HD-MUNE, CMAP, MUSIX	thenar	- progression detected (MUNE, MUNIX)
Cheah et al. (52)	3	37/0	CMAP, axonal excitability	abductor pollicis brevis	- progression detected (CMAP)
Ahn et al. (53)	NA	135/NA	NA	NA	- asymmetric progression (MUNE)
Cheah et al. (54)	3	58/NA	NI, CMAP	abductor digiti minimi and ulnar nerve	- progression detected (NI)
de Carvalho et al. (55)	6	28/0	NI, CMAP, MUNE	abductor digiti minimi muscles	- progression detected (CSP)
Neuwirth et al. (56)	15	7/8	MUNIX, CMAP,	abductor pollicis brevis (APB), abductor digiti minimi (ADM), abductor halluces brevis (AHB), extensor digitorum brevis (EDB)	- progression detected (MUNIX)
Floyd et al. (57)	18	60/33	TMS, CMCT, MEP	abductor digiti minimi (ADM) and tibialis anterior (TA)	-linear progression detected (TMS threshold, CMCT, TMS amplitude corrected)
Gooch et al. (58)	NA	64/NA-1	TMS, MUNE,	NA	-progression detected (MUNE)
Liu et al. (59)	12	112/12	MUNE, CMAP	Abductor pollicis brevis (APB) and abductor digiti quinti (ADQ)	- progression detected (MUNE), correlated to ALSFRS descent
Albrecht et al. (60)	11.5	10/25	MUNE, S-MUAP	extensor digitorum brevis	- progression detected (MUNE)
Wang et al. (61)	12	20/70	MUNE, SMUP, CMAP, MU loss	thenar	- progression detected - (Thenar MUNE, CMAP)
Chan et al. (62)	24	NA	motor units	thenar	- progression detected
Felice et al. (63)	12	NA	MUNE	thenar	- progression detected (MUNE)
Yuen et al. (64)	6	NA	CMAP, MUNE	abductor digiti minimi	- progression detected (MUNE, fiber density)
Vucic et al. (65)	7–100 days	25/30, 35	cortical and axonal excitability- MEP, CMAP- TMS	abductor pollicus brevis	- aim to determine effect of riluzole
Aggarwal et al. (66)	36	31/57	MUNE	tibialis anterior, abductor pollicis brevis (APB), deltoid, and first dorsal interosseous muscles	- no progression reported
Arasaki et al. (67)	NA	NA	MUNE,	extensor digitorum brevis (EDB)	- no progression reported
de Carvalho et al. (68)	11.6	NA	CMAP, MEP, TMS	NA	- no progression detected
Swash et al. (69)	NA	14/NA	single fiber EMG	NA	- no definite progression detected

**Table:** 

**Author(s) and year publication**	**Follow-up interval (months)**	**Number of patients/Number of controls**	**Clinical assessment batteries/Functional rating scales**	**Summary of findings**
**CLINICAL STUDIES**				
**ALSFRS-R**				
Thakore et al. (70)	NA	3367/0	ALSFRS-R, ALSFRS, bloods- creatinine, uric acid, CK, albumin, sodium bicarbonate, hematocrit, TWBC	- ALSFRS-R progression detected, pre-slope and post-slope have effects on survival
Rooney et al. (71)	NA	407/0	ALSFRS-R	- progression detected in ALSFRS-R subscores
^*^ACTS trial. (72)	NA	75/NA	ALSFRS	progression detected (ALSFRS-R), associated with motor and pulmonary function
**Cognitive and behavior assessments**				
Floeter et al. (73)	18	NA	ALSFRS-R, letter fluency, FBI	- progression detected (ALSFRS-R, FBI, letter fluency)
Elamin et al. (74)	NA	186/NA	cognitive testing	- progression detected (cognitive function)
Roberts-South et al. (75)	24	16/12	neuropsychology, language, discourse sampling, perfusion computerized transaxial tomography, pulmonary, clinical	- progression detected (cognitive language deficits)
^*^Duning et al. (76)	3	10/32	ALSFRS, clinical neuropsychological battery, imaging	- progression detected (DTI)
Poletti et al. (77)	24	168/0	ECAS	- no progression detected, ECAS scores improved over time
Xu et al. (78)	6	108/60	ACE-3, FAB, ECAS executive, MoCA, ALSFRS-R, ALS-FTD-Q, MiND-B	- no progression detected
Gillingham et al. (79)	9	20/36	ALS-CFB, ALSFRS-R	- no progression reported
Mioshi et al. (80)	6	79/53	MiND-B- apathy, disinhibition, stereotypical behavior, ACE-R, ALSFRS-R	- no progression reported
**Quality of life assessments**				
Jakobsson Larsson et al. (81)	24	36/0	SEIQoL-DW, ALSFRS-R, HADS	- anxiety decreased over time, depression correlated to QOL, QOL remained stable despite physical deterioration
**BMI and other clinical assessments**				
Beck et al. (82)	6	78/39	skin water loss	- progression detected (skin water loss)
Garruto et al. (83)	NA	31/66	bone mass (wrist radiograph)	- progression detected (bone loss)
Ioannides et al. (84)	6	44/29	FM-ADP, BMI, BAI, ALSFRS-R	- BMI and BAI not accurate measures of fat mass in ALS
Peter et al. (85)	3	393/791	BMI, ALSFRS-R	- alterations in body weight present in ALS patients decades before manifestation of symptoms
Nunes et al. (86)	3	37/0	BMI, serum albumin, transferrin, total cholesterol	- no progression reported
Jablecki et al. (87)	NA	NA	clinical scores	- no progression reported
**Respiratory and muscle assessments**				
Andres et al. (88)	4–21	100/0	ATLIS, ALSFRS, VC	- ATLIS more sensitive to change than ALSFRS and VC
de Bie et al. (89)	12	10/0	RSA, ALSFRS-R, FVC	- progression detected(RSA and ALSFRS-R)
Shellikeri et al. (90)	NA	33/13	kinematic measures of tongue and jaw movement, speaking rate, intelligibility, ALSFRS-R	- progression detected (tongue movement size and speed)
Londral et al. (91)	2–20	19/26	typing activity, ALSFRS-R	- progression detected (typing activity)
Panitz et al. (92)	12	51/0	fatigue severity scale (FSS), CIS20-R- subjective fatigue experience, concentration, motivation, activity, ALSFRS-R, MRC, SVC	- progression detected (FSS, CIS20-R), correlated to ALSFRS-R, and ALSFRS-R progression
Atassi et al. (93)	NA	8635/0	ALSFRS-R, VC	- PRO-ACT database- progression detected (ALSFRS-R and VC)
Watanabe et al. (94)	1.7 years	451/0	ALSFRS-R, MRC, MMT	-progression detected (ALSRS-R)
Leonardis et al. (95)	every 3 months	NA/0	ALSFRS-R, Norris-r, AGA, FVC, MIP, MEP, SNIP	- progression detected (respiratory measures)
Mahajan et al. (96)	NA	362/0	VC	- progression detected (VC)
Pinto et al. (97)	4–6	49/0	Diaphragm amplitude, ALSFRS-R, MIP, FVC, SNIP, SPO2	- progression detected (Diaphragm amplitude, ALSFRS-R, respiratory measures)
Montes et al. (98)	6	31/0	TUG, ALSFRS-R, FVC, MMT	- linear progression detected (TUG) - associated with ALSFRS-R, MMT
Vender et al. (99)	NA	139/0	FVC	- progression detected (FVC)
Wilson et al. (100)	NA	55/NA	respiratory- FVC, FEV1, PEFT	- linear progression detected (PEFT)
Poloni et al. (101)	NA	NA	VC, Motley index, FEV1	- progression detected (respiratory measures)
Andersen et al. (102)	6–59	20/0	respiratory- SVC, cough peak flow, max inspiratory muscle strength, SNIP, max insufflation capacity	- no progression reported
Quaranta et al. (103)	NA	NA	respiratory function	- no progression reported
Proudfoot et al. (104)	24	61/39	eye tracking- anti saccadic, trail making, visual search tasks, ALSFRS-R, ACE-R, UMN, imaging)	- no progression detected
^*^Lenglet et al. (105)	18	512/0	ALSFRS-R, MMT, SVC	- clinical trial
Yamauchi et al. (106)	Every 6 months	43/30	ALSFRS-R, phrenic nerve conduction study (DCMAP), respiratory function tests (SNIP, FVC), nocturnal pulsed oximetry, MMT	- no progression reported
Mendoza et al. (107)	NA	161/0	MIP, FVC	- no progression reported
Marti-Fabregas et al. (108)	NA	NA	FVC	- no progression detected
Palmowski et al. (109)	NA	NA	electro-oculography	- not well-defined progression

*Studies detecting progressive changes are listed first followed by studies not capturing longitudinal changes*.

* *indicates clinical trial*.

### Neurophysiology

Most longitudinal neurophysiology studies are single center studies, reducing the risk of inter-rater and inter-center variability (127). As presented in Table 1, follow-up interval ranges between 7 days (65) and 3 years (66), and up to 7 follow-up time-points have been included in some studies (57, 60). Surprisingly few studies include disease controls such as peripheral neuropathy (60) or benign fasciculation syndrome (50). Clinical assessments performed in conjunction with neurophysiology typically include ALSFRS-r (51), forced vital capacity (FVC) (55), slow vital capacity (SVC) (56), grip strength (64), pinch strength (58), and manual muscle testing (MMT) (58), however, correlations between neurophysiological measures and clinical assessments are seldom reported. The majority of longitudinal neurophysiological studies focus on upper limb muscles, e.g., abductor pollicis brevis, deltoid, first dorsal interrosseus, extensor digitorum brevis, abductor digiti minimi (51, 52, 55, 60, 61) with relatively few studies evaluating lower limb muscles such as abductor hallicus brevis and tibialis anterior (50, 56, 57, 66). The most commonly reported longitudinal neurophysiological indices include compound muscle action potential (CMAP) (51, 52), single motor unit action potential (SMUAP) (60), MUNE (55, 59), MUNIX (49, 56), neurophysiology index (NI) (54, 55), TMS measures (57, 58), and axonal excitability (52). Progressive neurophysiological changes have been detected by MUNIX (49, 51, 56), MUNE (51, 58, 60), CMAP (52, 61), NI (54), and TMS measures (57) and allowing for study-design limitations, the consensus is that degenerative changes are not linear.

### Clinical Biomarkers and Instruments

Robust clinical longitudinal studies in ALS have up to 6 follow-up time points (88, 89, 91), the interval between the assessments can be as short as 3 months (95) and the sample size can be as big as several thousands (70, 93) (Table 1). Few multi-timepoint studies include disease controls such as motor neuropathies (91), alternative neuromuscular diseases (78), or neurodegenerative conditions (83). Large, multi-timepoint longitudinal studies invariably suffer from considerable attrition rates, but these are rarely explicitly reported in the manuscript abstracts (10). Detailed genotyping is only available in a minority of longitudinal studies (15, 77, 79, 94). The most widely utilized rating scale in longitudinal studies is the ALSFRS-r (70, 71, 128) which provides a composite score of bulbar, limb and respiratory dysfunction, and is invariably evaluated in clinical trials (72, 105). Quality of life (QoL) in ALS is increasingly evaluated by disease-specific instruments such as the 40-item ALS assessment questionnaire (ALSAQ-40) or the revised ALS-specific Quality of Life questionnaire (ALSSQoL-R) (129–131). A number of symptom-specific instruments are also commonly used such as the Center for Neurologic Study-Bulbar Function Scale (CNS-BFS), a 21-item self-report scale of bulbar function, and the Center for Neurologic Study-Lability Scale (CNS-LS), a 7-item self-report scale of pseudobulbar affect (PBA) (132). Tapping rates, composite reflex scores, The Penn UMN Score (133), the Modified Ashworth scale (MAS) are often used as proxies of UMN degeneration (132).

In clinical trials, muscle strength is often estimated by handheld dynamometry (HHD) (134), manual muscle testing (MMT) (105), scoring systems such as the Medical Research Council (MRC) Scale for muscle strength (135) and some studies also report limb circumference (136). Respiratory function in ALS is typically monitored by sniff nasal inspiratory pressure (SNIP), SVC, or FVC in addition to measures such as early morning arterial blood gas (ABG) and overnight pulse-oximetry (137, 138). Measures of typing ability (91), tongue movements (90), vital capacity (VC) (96), FVC (99), SNIP (97), and diaphragm amplitude (97) all show progressive longitudinal changes. Nutritional markers such as body mass index (BMI) and lipid profile are now established prognostic indicators (139, 140). Cognitive and behavioral domains are routinely assessed thanks to the availability of validated screening instruments such as the Edinburgh Cognitive and Behavioral ALS Screen (ECAS) (141), the Beaumont Behavioral Inventory (BBI) (142) and the ALS Cognitive Behavioral Screen (ALS-CBS) (143). In contrast to the relentlessly progressive motor deficits of ALS, the trajectory of cognitive and behavioral deficits is less clear due to considerable individual variations, genotype-associated profiles (144, 145), differences in assessment strategies and practice-effects (146). Several longitudinal neuropsychology studies do not detect progression (77, 147, 148), progressive behavioral impairment has been noted in the absence of cognitive change (149), and some studies report improved performance as a result of practice effects (77).

### Wet Biomarkers

The findings, study design characteristics, and follow-up intervals of longitudinal biofluid studies are summarized in Table 2. Phosphorylated neurofilament heavy chain (pNFH), neurofilament light chain (NF-L), progranulin (PGRN), cytokines, TAR DNA-binding protein 43 (TDP-43), cystatin C, creatinine, micro-RNAs (miRNAs), chitotriosidase-1 (CHIT1), chitinase-3-like protein 1 (CHI3L1), chitinase-3-like protein 2 (CHI3L2) have been evaluated in both research studies (152, 153, 157, 158, 162, 164, 168, 171) and clinical trials (150, 156, 157, 160, 161). Markers of iron metabolism and ferroptosis are relatively recent domains of ALS biomarker research (172, 173). Most biofluid studies are either serum (150, 157) or CSF studies (152, 167), but urine (155) and skeletal muscle-based (153) studies have now also been published. Quantitative enzyme-linked immunosorbent assay (ELISA) is the most commonly used antibody-based technique (13, 174) which can be performed with one antibody (indirect ELISA), or with two antibodies (sandwich ELISA). Increased CSF (13) and serum (175) pNFH detected by ELISA is thought to be a sensitive marker of axonal degeneration in ALS (152, 171, 176, 177). The specificity of this marker however may be inadequate to reliably differentiate ALS from other neurodegenerative conditions (13, 176). Other antibody-based techniques such as Western blot (171) and electrochemiluminescence (ECL) (153, 168) may improve detection sensitivity and reliability (13). Panels of multiple proteins can be evaluated by multiplex immunoassays such as planar or microbead assays (13). Mass spectrometry based methods using chromatin-immunoprecipitation-based surfaces, two-dimensional gel electrophoresis or high-resolution mass spectrometry have identified cystatin-C and transthyretin as candidate biomarkers (178–180). The longest wet biomarker study followed patients for 4 years (164). The majority of studies have at least 2 follow-up timepoints (155, 162, 170) and one study included 13 follow-up timepoints (156, 159). Large multi-center trials include as much as 1,000 participants (156). One of the most striking shortcomings of existing longitudinal studies is that very few included disease controls such as Parkinson's disease cohorts, patients with multifocal motor neuropathy with conduction block, Kennedy's disease, chronic inflammatory demyelinating polyneuropathy (CIDP), cervical or lumbar radiculopathy, Charcot-Marie-Tooth disease (CMT), benign fasciculation, and cramp syndrome etc. (152, 159, 162). Another limitation of many longitudinal studies is the lack of comprehensive genotyping (12) as very few studies report comprehensive screening for ALS-associated mutations (153, 159, 169, 171). Exhaustive clinical profiling, such as medications (152, 164), neuropsychological assessments (171), quality of life indices are rarely reported in longitudinal studies. The majority of studies limit their clinical descriptions to ALSFRS-r, FVC, MRC, and Ashworth scores (153, 161, 162). Serum and plasma biomarkers such as creatinine (150, 156), pNfH (158, 159), and micro-RNAs (157), CSF biomarkers such as CHI3L1 (152), tau (160, 161), and cystatin-C (162), and urinary (155) and skeletal muscle (153) biomarkers are some of the promising tools for detecting disease progression. While no progressive changes have been detected in NFL levels, it is likely to be a useful as a diagnostic biomarker (168, 171).

**Table 2:** Longitudinal “wet biomarker” studies in ALS.

**Author(s) and year of publication**	**Follow-up interval (months)**	**Number of patients / number of controls**	**Candidate biomarker evaluated**	**Biofluid**	**Assessment method used**	**Summary of conclusion**
^*^Okada et al. (150)	12	57/0	creatinine	serum	NA	-progression detected (creatinine)
Raheja et al. (151)	NA	NA	microRNAs	serum	NA	- progression detected (miR-136-3p, miR-30b-5p, miR-331-3p, miR-496, miR-2110)
Thompson et al. (152)	30	49/52	chitotriosidase (CHIT1), chitinase-3-like protein 1 (CHI3L1), and chitinase-3-like protein 2 (CHI3L2), (phosphorylated neurofilament heavy chain) Pnfh	CSF	nano ultra-high performance liquid chromatography tandem mass spectrometry (nUHPLC LC-MS/MS), ELISA	- progression detected (CHI3L1)
Di Pietro et al. (153)	NA	14/24	micro-RNAs- MIR206, MIR208B, MIR499	skeletal muscle	quantitative real time PCR, Western blot analysis	- progression detected (MIR208B, MIR499, MIR206, HDAC4)
Murdock et al. (154)	Every 6–12 months	119/35	leukocytes	blood	flow cytometry	- progression detected (immune cells), associated with ALSFRS-R
Shepheard et al. (155)	NA	54/45	urinary p75ECD	urine	sandwich ELISA	- progression detected (urinary p75ECD), correlated with ALSFRS-R
van Ejik et al. (156)	NA	1241/0	creatinine	plasma	NA	- progression detected (plasma creatinine), correlated to ALSFRS-R, muscle strength, mortality
Waller et al. (157)	3	22/0	microRNAs, miR-17-5p, miR-223-3p, miR-24	serum	Qiagen miScript-based Qpcr	- progression detected (mir-206, mir-143-3p, mir-374b-5p)
McCombe et al. (158)	27	98/61	pNFH	serum	NA	- progression detected (pNFH)
Lu et al. (159)	36	136/104	neurofilament heavy chain-phosphoform	plasma	ELISA	- progression detected (NfH)
^*^Levine et al. (160)	6	28/0	tau, pNFH	CSF	ELISA	- progression detected (tau)
^*^Levine et al. (161)	12	20/0	tau, pNFH	CSF	ELISA	- progression detected (tau)
Wilson et al. (162)	24	44/60	cystatin C	CSF, plasma	quantitative enzyme linked immunosorbent assay (ELISA)	- progression detected (cystatin C)
Gaiani et al. (163)	36	94/82	ALSFRS-R, NFL	CSF	enzyme-linked immunosorbent assay (UmanDiagnostics AB)	- NFL may have role as a biomarker
Lu et al. (164)	48	95/88	CK, ferritin, tumor necrosis factor (TNF)–a, and interleukin (IL)−1b, IL-2, IL-8, IL-12p70, IL-4, IL-5, IL-10, and IL-13, IL-6, IFN-Y	plasma	multiplex electrochemiluminescence immunoassay	- no defined progression
Steinacker et al. (165)	24	125/28	neurofilament light chain (NF-L), progranulin (PGRN), S100	serum, CSF (baseline only)	ELISA, electrochemiluminescence (ECL) immunoassay, ECLIA Elecsys (Roche, Penzberg, Germany)	- no progression reported
Gibson et al. (166)	12	80/0	CK	NA	NA	- no progression detected
Gray et al. (167)	24	41/14	CSF- glucose, lactate, citric acid, ethanol	CSF	H-NMR	- no progression reported
Lu et al. (168)	36	167/78	neurofilament light chain (NFL)	serum, blood, CSF	electrochemiluminescence immunoassay	- no progression detected
Verstraete et al. (169)	NA	219/100	TDP-43	plasma	sandwich ELISA	- no defined progression
Nardo et al. (170)	6	94/64	PRDX2, GSTO1, CLIC1, HSC70, CypA, PDI, ERp57, CALR, PA28a, IRAK4, FUBP1, ROA2, actinNT, TDP-43	blood PBMC	2D-DIGE, mass spectometry	- no progression reported

* *indicates clinical trial*.

### Studies of Asymptomatic Mutation Carriers

Current clinical trials only recruit symptomatic cases despite accruing evidence that ALS has a long presymptomatic phase (5). Imaging studies of asymptomatic mutation carriers have consistently confirmed disease-specific cerebral and spinal cord changes prior to symptom onset (181–184) indicating that this disease-phase may represent a crucial window for therapeutic or neuroprotective intervention. The majority of presymptomatic studies assess a single time-point, as opposed to the longitudinal tracking of asymptomatic carriers of ALS-causing mutations (15). While the overwhelming majority of presymptomatic studies focus on *C9orf72* hexanucleotide carriers (183, 185–187), no prognostic markers have been validated to predict whether single patients will develop ALS or FTD. Compared to imaging studies, strikingly few presymptomatic neurophysiology studies have been undertaken (66). Studies of asymptomatic ALS-causing mutation carriers have enormous potential for academic research and may pave the way for asymptomatic pharmaceutical trials (5, 181).

## Discussion

Clinical trials currently evaluate the efficacy of candidate drugs using the revised ALS functional rating scale (ALSFRS-r), muscle strength assessment tools such as manual muscle testing (MMT), respiratory function indices such as forced vital capacity (FVC), slow vital capacity (SVC) and sniff nasal inspiratory pressure (SNIP), neurophysiological measures and survival (102, 116, 120, 188, 189). These measures however primarily reflect late-stage functional impairment and are not indicative of early stage pathology. Brain and spinal cord imaging has been evaluated as early-stage biomarkers with both diagnostic and monitoring potential (116, 120, 190). The core neuroimaging signature of ALS, irrespective of the disease-stage, includes corticospinal tract (191, 192), corpus callosum (193) and motor cortex degeneration (194). Atrophy in frontotemporal regions has been primarily associated with neuropsychological deficits (195–197) and linked to hexanucleotide repeats in C9orf72 (145, 198). Longitudinal imaging studies are superior to cross-sectional studies as they readily detect dynamic structural and functional changes and may elucidate compensatory processes (10, 14, 23, 28, 40, 120, 199). The emergence of multi-timepoint study designs (14, 20) enable the characterization of anatomical propagation patterns (200) and provide invaluable temporal insights into the disease trajectory of late-stage ALS. Inter-scan intervals as short as 3 months can detect longitudinal changes (14, 18, 120). Many longitudinal studies make use of multiple magnetic resonance (MR) metrics which is particularly useful in establishing an optimal panel of monitoring markers (120). Several longitudinal studies have indicated that white matter degeneration can be detected relatively early in the course of ALS with restricted further progression over time, whereas gray matter pathology shows relentless progression in the symptomatic phase of the disease (4, 14, 120). In addition to structural imaging studies, connectivity-based, metabolic, peripheral nerve, and, whole body muscle imaging have contributed to our understanding of longitudinal changes (20, 201–203).

Needle electromyography and nerve conduction studies play an important clinical role in ruling out alternative conditions and confirming a suspected diagnosis of ALS. Despite variations in local protocols, neurophysiological tests are recognized as objective, reliable and cost-effective tests of neuromuscular dysfunction, and have also been repeatedly proposed as longitudinal markers (55, 204). CMAP is generated by depolarization of muscle fibers through the stimulation of a single nerve, where amplitude reductions are interpreted as loss of motor axons (205, 206). While CMAP measurements capture longitudinal decline, it is confounded by variations in temperature, limb positioning and electrode placement (56, 207). CMAP-derived measures such as MUNE and MUNIX are now extensively utilized to characterize progressive changes in ALS. MUNE estimates motor neuron numbers, and may detect the rate of motor neuron loss, making it a more reliable method of appraising disease progression than CMAP (208, 209). However, its early-phase sensitivity has been questioned, as its use is limited to distal muscles, and the technique requires considerable training, especially for inter-rater and multi-site comparisons (205, 210). TMS allows the characterization of upper motor neuron dysfunction, and may be particularly useful in detecting progressive changes (57, 205).

Functional rating-scales are often the monitoring instruments of choice in clinical trials (55), as they are easy to administer, cost-effective to utilize and have acceptable inter- and intra-rater reliability profiles (7). The most widely used rating scale in clinical longitudinal studies is the ALSFRS-r. Despite its ease of administration, it has considerable limitations, as it may be disproportionately influenced by LMN dysfunction, does not account for laterality or asymmetry of symptoms, omits cognitive impairment, and may be affected by medications (14, 128, 188, 211).

Proteomics, metabolomics and lipidomics have seen significant advances in ALS research and CSF and serum markers are now used in longitudinal academic and pharmacological studies (172). Potential biomarkers for the detection of disease progression include serum and plasma biomarkers such as creatinine (150, 156), pNfH (158, 159), and micro-RNAs (157), CSF biomarkers such as CHI3L1 (152), tau (160, 161), and cystatin-C (162), and urinary (155) and skeletal muscle (153) biomarkers.

### Prediction Analyses

Age at symptom onset (212), BMI (139), bulbar involvement (213), cognitive impairment (214), *C9orf72* genotype status (144), respiratory insufficiency (215), “definite ALS” by the El Escorial criteria (216), and functional disability (217) are the most commonly cited determinants of poor prognosis in ALS. SNIP (218) and less commonly used measures such as twitch trans-diaphragmatic pressure (Tw Pdi) (219) and maximal static expiratory mouth pressure (MEP) were shown to be good predictors of ventilator-free survival (219). A combined panel of several clinical, wet, and dry biomarkers is likely to offer the most accurate prognostic information (115, 120, 216, 217, 220). While cerebral (217, 221, 222) and spinal (115) imaging measures have been repeatedly linked to survival outcomes, these have not been utilized in a clinical setting. Neurophysiological variables, such as phrenic nerve stimulation outcomes (223) and biofluid markers, such as pNFH and NFL (165, 168, 224–226) are also thought to be accurate predictors.

### Patient Stratification

Attempts to enroll patients in the early stages of the disease are hampered by the universally long diagnostic delay in ALS (227). Patient stratification in trials is typically based on site of onset (228), instead of other variables which have an established prognostic impact (138, 229). Admixed patient cohorts within a trial may hamper the ability to detect how different phenotypes and genotypes may exhibit a different response to a candidate drug (230–232). The stratification of heterogeneous cohorts is now aided by the development of validated staging systems, such as the King's (233), Milano-Torino (MITOS) (234) or the Fine'til 9 (FT9) (235) staging systems. The King's Staging system is based on the number of body regions affected, and the presence of nutritional or respiratory failure (233). The MITOS staging system is based on the ALSFRS-r, and is particularly sensitive to changes in later stages of the disease (236, 237). However, none of these staging systems account for cognitive or behavioral changes (236). Pathological staging systems suggest a four-stage model of ALS based on anatomical patterns of pTDP-43 load (238, 239). This system has now been validated by *in vivo* neuroimaging studies (240) and signals that accurate pathological staging and patient stratification may be possible based on neuroimaging (199, 240).

### International Consortia

Only few ALS centers maintain dedicated biobanking facilities to store and process molecular markers in human biofluid locally. Similarly, relatively few centers are in a position to generate sufficient number of MRI and neurophysiology data sets of rare phenotypes to make meaningful inferences in a single center setting. Brain and tissue banks are also challenging to establish, maintain and fund, despite their invaluable contribution to ALS research (241–243).

Biospecimen samples are also often collected during clinical trials, and discarded after negative outcomes, despite their enormous potential for biomarker discovery (172). One of the most important achievements of biomarker development efforts is the establishment of national and international research consortia such as Association pour la recherche sur la SLA (ARSLA), Neuroimaging Society in ALS (NISALS), Research Motor Neuron (RMN), Canadian ALS Neuroimaging Consortium (CALSNIC), EU Joint Programme for Neurodegenerative Disease Research (JPND), European multidisciplinary ALS network identification to cure motor neurone degeneration (EUROMOTOR) which maintain vital biobanking facilities, registries, data repositories for multicenter data interpretation (121, 244). Clinical trial networks are also increasingly recognized as valuable platforms for multisite data collection and interpretation as they operate with carefully standardized protocols. Consortia such as the European Registry of ALS (EURALS) Consortium, the Western ALS (WALS) Consortium and the Northeast ALS (NEALS) Consortium are other examples (245). NEALS is one of the largest consortia with over 100 member sites from the US, Canada, Mexico, Italy, Lebanon and Australia (246). EURALS coordinates research studies and clinical trials relying on population-based European registries and include centers from Scotland, England, Netherlands, Spain, Ireland, Serbia, Italy, France, and Germany (241, 247, 248). ALS research consortia promote patient-oriented research, maintain biofluid, imaging and DNA banks, and have the potential to translate scientific advances into pragmatic clinical interventions.

### Telehealth

Novel trends in longitudinal data collection include telemedicine-based technologies, wearable sensors and mobile phone applications (230). The continuous collection of data via telephone or telemedicine applications such as the Telehealth in Motor Neuron disease (TiM) system circumvent the inconvenience of patients and caregivers traveling long distances for research appointments (249). Once local data-protection and governance guidelines are complied with, information uploaded from these systems can be made available to healthcare professionals of multidisciplinary teams in real time (249). The feasibility of telehealth for ALS patients via live video-conferencing has also been evaluated (250) and is considered a particularly promising clinical and research platform (249, 250). A number of cognitive-behavioral screening tools have also been adapted for phone administration (251) including modified versions of the ALS Cognitive Behavior Screen (ALS-CBS), the Controlled Oral Word Association Test (COWAT), the Center for Neurologic Study-Lability Scale (CNS-LS) and found to be statistically equivalent to face-to-face assessments (251). Performance on other tests however, such as the telephone versions of the ALS-Frontal Behavioral Inventory (ALS-FBI) caregiver interview and the Written Verbal Fluency Index (WVFI) was not equivalent to clinic-based assessments (251). The continued development of telephone and internet-enabled devices are likely to provide further insights to longitudinal physical, cognitive and behavioral changes (251).

## Conclusions

While clinical indicators of disease progression remain indispensable, neuroimaging, neurophysiology, and biofluid measures are particularly promising, objective, quantitative biomarker candidates. The validation of combined “wet” and “dry” biomarker panels will not only enable the detection of subtle progressive changes in ALS, but allow precision stratification of heterogeneous patient cohorts in clinical trials and improve existing prediction algorithms.

## Author Contributions

The manuscript was drafted by RC. EF, SL, OH and PB contributed to the conceptualization, editing, and revision of this paper.

### Conflict of Interest Statement

The authors declare that the research was conducted in the absence of any commercial or financial relationships that could be construed as a potential conflict of interest.

## Abbreviations

2D-DIGE: two-dimensional fluorescence difference gel electrophoresis
ABG: arterial blood gas
ACE-3: Addenbrookes Cognitive Examination - Third Edition
ACE-R: Addenbrooke's Cognitive Examination-revised
AD: axial diffusivity
ADM: abductor digiti minimi
ADQ: abductor digiti quinti
AGA: arterial gas analyses
AHB: abductor halluces brevis
ALS: amyotrophic lateral sclerosis
ALSAQ-40: ALS assessment questionnaire
ALS-CBS: ALS Cognitive Behavior Screen
ALS-CFB: ALS computerized frontal battery
ALS-FBI: ALS-Frontal Behavioral Inventory
ALSFRS-r: revised ALS functional rating scale
ALSS: ALS severity scale
ALSSQoL-R: revised ALS-specific Quality of Life questionnaire
APB: abductor pollicis brevis
ARSLA: Association pour la recherche sur la SLA
ATLIS: accurate test of limb isometric strength
BAI: body adiposity index
BMI: body mass index
CALR: Calreticulin
CALSNIC: Canadian ALS Neuroimaging Consortium
CHI3L1: chitinase-3-like protein 1
CHI3L2: chitinase-3-like protein 2
CHIT1: chitotriosidase-1
Cho: Choline
CIDP: chronic inflammatory demyelinating polyneuropathy
CIS20-R: checklist individual strength
CK: creatinine kinase
CLIC1: Chloride intracellular channel protein 1
CMAP: compound muscle action potential
CMCT: central motor conduction time
CMT: Charcot-Marie-Tooth disease
CNS-BFS: Center for Neurologic Study-Bulbar Function Scale
CNS-LS: Center for Neurologic Study-Lability Scale
COWAT: controlled oral word association test
Cr: creatinine
CSF: cerebrospinal fluid
CSP: cortical silence period
CypA: peptidyl-prolyl cis-trans isomerase A
DCMAP: distal compound muscle action potential
D-KEFS: Delis–Kaplan Executive Function System
DTI: diffusion tensor imaging
DWI: diffusion-weighted imaging
ECAS: Edinburgh Cognitive and Behavioral ALS Screen
ECL: electrochemiluminescence
EDB: extensor digitorum brevis
EMG: electromyography
ERp57: protein disulfide-isomerase A3
EURALS: European Registry of ALS Consortium
EUROMOTOR: European multidisciplinary ALS network identification to cure motor neurone degeneration
FA: fractional anisotropy
FAB: frontal assessment battery
FBI: frontal behavioral inventory
FD: fiber density
FEV1: forced expiratory volume
fibs-sw: fibrillation/sharp-waves
FM-ADP: fat mass air displacement plethysmography
FPs: fasciculation potentials
FSS: fatigue severity scale
FUBP1: far upstream element-binding protein 1
FVC: forced vital capacity
GM: gray matter
GSTO1: glutathione S-transferase omega-1
HADS: hospital anxiety and depression scale
HDAC4: histone deacetylase 4
HHD: handheld dynamometry
HSC70: heat shock cognate 71 kDa protein
IL: interleukin
IFN: interferon
IRAK4: Interleukin-1 receptor-associated kinase 4
JPND: EU Joint Programme for Neurodegenerative Disease Research
LGVF: letter guided verbal fluency
LMN: lower motor neuron
MAS: modified Ashworth scale
McDESPOT: multi-component driven equilibrium single pulse observation of T1/T2
MDRS-2: Mattis Dementia Rating scale-Second Edition
MEP: maximal static expiratory mouth pressure
MiND-B: motor neuron disease behavior scale
MIP: maximal inspiratory pressure
MiRNAs: micro-RNAs
MITOS: Milano-Torino staging system
MMSE: mini mental state examination
MMT: manual muscle testing
MND: Motor neuron disease
MoCA: Montreal Cognitive Assessment
MR: magnetic resonance
MRC: Medical Research Council Scale for muscle strength
MRCSS-LL: Medical Research Council sum score
MRI: magnetic resonance imaging
MRS: magnetic resonance spectroscopy
MS: multiple sclerosis
MU: motor unit
MUNE: motor unit number estimation
MUNIX: motor unit number index
MUPs: motor unit potentials
MUSIX: motor unit size index
NAA: N-acetylaspartate
NEALS: Northeast ALS Consortium
NF-L: neurofilament light chain
NI: neurophysiology index
NISALS: Neuroimaging Society in Amyotrophic Lateral Sclerosis
NMR: nuclear magnetic resonance
nUHPLC LC-MS: nano ultra-high performance liquid chromatography tandem mass spectrometry
p75ECD: neurotrophin receptor p75 extracellular domain
PA28a: proteasome activator complex subunit 1
PBA: pseudobulbar affect
PCR: polymerase chain reaction
PDI: protein disulfide-isomerase
PEFT: peak expiratory flow time
PET: positron emission tomography
PGGM: precentral gyruses gray matter
PGRN: progranulin
PLS: primary lateral sclerosis
PMA: progressive muscular atrophy
pNFH: Phosphorylated neurofilament heavy chain
PRDX2: peroxiredoxin-2
PRO-ACT: Pooled Resource Open-Access ALS Clinical Trials
QoL: quality of life
RD: radial diffusivity
RMN: Research Motor Neuron
ROA2: Heterogeneous nuclear ribonucleoproteins A2/B1
RSA: relative surface area
rsfMRI: resting state functional magnetic resonance imaging
SCA: spinocerebellar ataxia
SBMA: spinal and bulbar muscular atrophy
SEIQOL-DW: Schedule for the Evaluation of the Individual Quality of Life-Direct Weighting
SF-36: 36-Item short form health survey
SMA: spinal muscular atrophy
SMUAP: single motor unit action potential
SNIP: sniff nasal inspiratory pressure
SOD1: superoxide dismutase 1
SOP: Standard operating procedure
SPECT: single photon emission computed tomography
SPO2: peripheral capillary oxygen saturation
SVC: slow vital capacity
TA: tibialis anterior
TDP-43: TAR DNA-binding protein 43
TiM: Telehealth in Motor Neuron disease
TMS: transcranial magnetic stimulation
TNF: tumor necrosis factor
TUG: timed up and go test
Tw Pdi: twitch trans-diaphragmatic pressure
TWBC: total white blood cell count
UMN: upper motor neuron
VC: vital capacity
WALS: Western ALS Consortium
WVFI: Written Verbal Fluency Index.
